# Development of a genetic engineering toolbox for syngas-utilizing acetogen *Clostridium* sp. AWRP

**DOI:** 10.1186/s12934-023-02272-2

**Published:** 2024-01-03

**Authors:** Hae Jun Kwon, Joungmin Lee, Soo Jae Kwon, Hyun Sook Lee

**Affiliations:** 1 Marine Biotechnology Research Center, Korea Institute of Ocean Science and Technology, Busan, Republic of Korea; 2 KIOST School, University of Science and Technology, Busan, Republic of Korea

**Keywords:** *Clostridium* sp. AWRP, Acetogen, Transformation, Genome editing, CRISPR/Cas12a

## Abstract

**Background:**

*Clostridium* sp. AWRP (AWRP) is a novel acetogenic bacterium isolated under high partial pressure of carbon monoxide (CO) and can be one of promising candidates for alcohol production from carbon oxides. Compared to model strains such as *C. ljungdahlii* and *C. autoethanogenum*, however, genetic manipulation of AWRP has not been established, preventing studies on its physiological characteristics and metabolic engineering.

**Results:**

We were able to demonstrate the genetic domestication of AWRP, including transformation of shuttle plasmids, promoter characterization, and genome editing. From the conjugation experiment with *E. coli* S17-1, among the four replicons tested (pCB102, pAMβ1, pIP404, and pIM13), three replicated in AWRP but pCB102 was the only one that could be transferred by electroporation. DNA methylation in *E. coli* significantly influenced transformation efficiencies in AWRP: the highest transformation efficiencies (10^2^–10^3^ CFU/µg) were achieved with unmethylated plasmid DNA. Determination of strengths of several clostridial promoters enabled the establishment of a CRISPR/Cas12a genome editing system based on *Acidaminococcus* sp. BV3L6 *cas12a* gene; interestingly, the commonly used CRISPR/Cas9 system did not work in AWRP, although it expressed the weakest promoter (*C. acetobutylicum* P_*ptb*_) tested. This system was successfully employed for the single gene deletion (*xylB* and *pyrE*) and double deletion of two prophage gene clusters.

**Conclusions:**

The presented genome editing system allowed us to achieve several genome manipulations, including double deletion of two large prophage groups. The genetic toolbox developed in this study will offer a chance for deeper studies on *Clostridium* sp. AWRP for syngas fermentation and carbon dioxide (CO_2_) sequestration.

**Supplementary Information:**

The online version contains supplementary material available at 10.1186/s12934-023-02272-2.

## Introduction

Biorefinery has emerged as a way to reduce dependency on fossil fuels by producing alternative fuels and commodity chemicals from renewable biomass [1, 2]. However, the use of edible feedstocks should be restricted as much as possible since it provokes debates such as the “food vs. fuel dilemma” [3]. Therefore, the use of nonedible biomass including lignocellulose, oil, and algal biomass has been focused on for the past decades [2, 4]. More recently, direct utilization of C1 gases (i.e., CO_2_, CO, and CH_4_) is receiving great attention, as such gases can be synthesized through gasification of recalcitrant biomass and waste from human activities [5, 6]. Acetogenic bacteria use the Wood-Ljungdahl pathway (WLP) for their autotrophic growth, through which carbon monoxide (CO) or dioxide (CO_2_) is assimilated to acetyl-CoA [7]. WLP is distinguished from other carbon fixation pathways for its low demand for ATP and its ability to assimilate CO as a carbon and energy source [8].

Among the acetogenic bacteria, *Clostridium ljungdahlii* and *C. autoethanogenum* have been subjected as model strains to studies aimed at industrial applications as they are able to produce ethanol and 2,3-butanediol in addition to acetate [9–11]. Genetic tools for these clostridial strains have been increasingly expanded, including the mobile group II intron [12], allele-coupled exchange (ACE) [13] and CRISPR/Cas-based genome editing [14–16]. However, these tools have seldom been exploited in other nonmodel strains. The lack of solid genetic manipulation tools is a serious impediment to studies on bacteria with phenotypes different from those of model strains, which are important resources for understanding the metabolism of acetogenic bacteria, especially alcohol production.

*Clostridium* sp. AWRP is an alcohol-producing acetogenic bacterium that was isolated in a wetland [17]. From previous studies, this bacterium appears to exhibit a different alcohol production metabolism compared to model strains: its ethanol selectivity to acetate could be greatly increased by CO supply [17, 18]. Nonetheless, in this strain, there are several questions that remain unclear including its nutritional requirements [19], which genetic modifications may be required to address. Therefore, we sought to develop procedures for genetic modifications in *Clostridium* sp. AWRP, from transformation to CRISPR-based genome editing. Through the genome editing system proposed, it was possible to not only delete a single gene but also attain deletion of two large prophage clusters from the AWRP genome, showing that the system can be useful for various studies, including the physiological and metabolic engineering of this bacterium.

## Results and discussion

### Plasmid transfer to *Clostridium* sp. AWRP

**Fig. 1:**
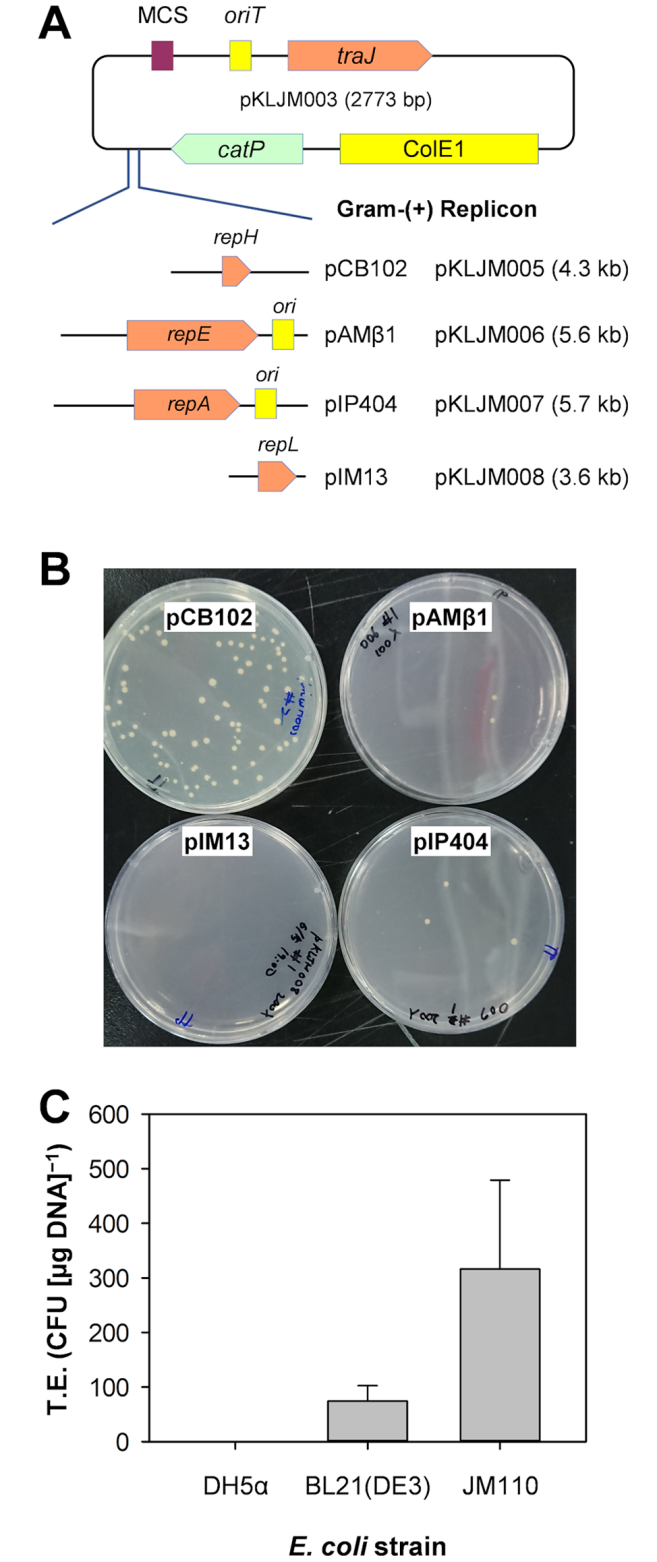
Transfer of shuttle plasmids into *Clostridium* sp. AWRP. (**A**) Construction of shuttle plasmids with various gram-positive replicons. (**B**) Conjugal transfer of the shuttle plasmids. (**C**) Transformation efficiencies (T.E.) of *Clostridium* sp. AWRP with pKLJM005 isolated from *E. coli* strains with different DNA methylation patterns

The introduction of foreign DNA, including episomal vectors, is a crucial step in the development of genetic tools for a strain. As it is already known that *C. ljungdahlii* and *C. autoethanogenum* are transformable with various shuttle plasmids, we expected that the transformation of *Clostridium* sp. AWRP (hereafter referred to as AWRP) might not be a major bottleneck, considering AWRP is phylogenetically close to the two strains [17]. However, our attempts to transform AWRP with shuttle plasmids harboring various gram-positive replicons via electroporation were unsuccessful, despite modifications to cell growth phase, electroporation buffer, and electric parameters. It was not clear whether the failure of transformation resulted from the replicon. Therefore, we attempted to introduce these plasmids into AWRP via biparental conjugal transfer, which has been widely applied to various *Clostridium* species that are not easily transformed by electroporation [20, 21]. To transfer various gram-positive replicons to AWRP, we first constructed a compact backbone plasmid pKLJM003, which contained a high-copy ColE1 replicon for *E. coli* (from pMTL500E), the chloramphenicol/thiamphenicol resistance marker, and RK2/RP4 *traJ-oriT*, which is necessary for plasmid mobilization (see Table S1). Four shuttle plasmids, pKLJM005 to 008, were constructed by cloning different gram-positive replicons: pCB102 (from *C. butyricum*), pAMβ1 (*Enterococcus faecalis*), pIP404 (*C. perfringens)* and pIM13 (*Bacillus subtilis*), respectively (Fig. 1A). Among four replicons tested, we found that the pCB102 replicon (in pKLJM005) was the most efficiently transferred to AWRP, although with very low efficiencies (approximately 10^− 8^ transconjugants per donor cells; Fig. 1B). The replicons pAMβ1 and pIP404 were also found to be transferred to AWRP but yielded fewer colonies than pCB102 (< 10^− 9^ transconjugants per donor; Fig. 1B). In contrast to previous studies on *C. ljungdahlii* [10, 22, 23], pKLJM008 (pIM13 replicon) was not introduced into AWRP (Fig. 1B). Therefore, the plasmid pKLJM005 was chosen to establish the electrotransformation method for AWRP.

Next, we determined the presence of restriction endonuclease activities, which are known to be the major barriers to DNA transfer in various clostridial strains [24–26]. However, no evidence of DNA digestion was observed when pKLJM005 was treated with the crude extract of AWRP cells and commercially available restriction enzyme buffers, indicating that recalcitrance of AWRP may not result from the existence of typical type II restriction endonucleases (data not shown). DNA methylation patterns generated by *E. coli* Dam and Dcm methyltransferases have been reported to affect the transformation efficiency in *C. ljungdahlii* for an unknown reason [22, 23]. Therefore, we performed the transformation of AWRP with pKLJM005 plasmids isolated from three strains of *E. coli*: DH5α, BL21(DE3), and JM110, which are *dam*^+^/*dcm*^+^, *dam*^+^/*dcm*^−^, and *dam*^−^/*dcm*^−^, respectively. Dam and Dcm methylation were found to have a negative impact on AWRP’s transformation efficiency (Fig. 1C). The unmethylated plasmid yielded the highest transformation efficiency (3.2 ± 1.6 × 10^2^ cfu [µg DNA]^−1^), and the Dam-methylated plasmid exhibited lower efficiencies (74 ± 28 cfu [µg DNA]^−1^; Fig. 1C). However, all attempts to introduce Dam/Dcm-methylated DNA were unsuccessful. Based on the result, we concluded that Dam and Dcm methylation have an adverse impact on the transformation efficiencies of AWRP as in *C. ljungdahlii*, with Dcm methylation likely the major barrier; pKLJM005 has 10 Dam methylation sites and 8 Dcm methylation sites. As previously mentioned, we did not observe restriction enzyme activities in vitro, and the reason why unmethylated DNA has been effectively introduced is unclear. In REBASE from New England Biolabs (http://rebase.neb.com), it is predicted that two putative restriction endonucleases are encoded in the AWRP genome: Type I (DMR38_11115-11130), which requires ATP for its activity, and Type IIG (DMR38_15120), which requires *S*-adenosylmethionine. Although the activities of these endonucleases were not identified in this study, these genes can be deletion targets for achieving higher transformation efficiencies [27].

Notably, pKLJM006 and pKLJM007, which were successfully transferred to AWRP via conjugation, were not introduced into AWRP via electroporation, although they were prepared from *E. coli* JM110 (data not shown). Therefore, the plasmids used in the following experiments were constructed using the pCB102 replicon.

### Evaluation of promoters for gene expression

**Fig. 2:**
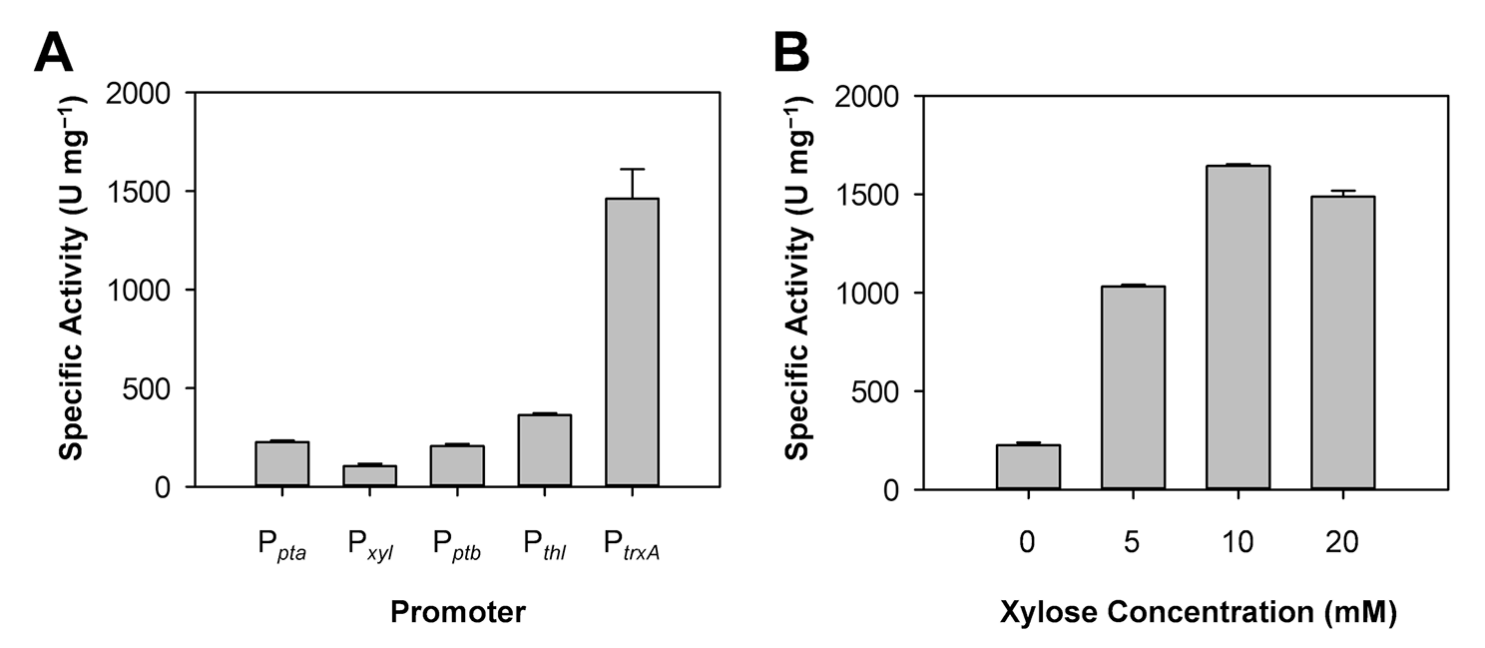
Determination of promoter strengths in *Clostridium* sp. AWRP. (**A**) Comparison of the strengths of different promoters in *Clostridium* sp. AWRP. The crude extract of wild-type AWRP did not show background β-galactosidase activities and is not shown. The value of *P*_*xyl*_ was obtained without supplementation with xylose. (**B**) Strengths of P_*xyl*_ with various concentrations of xylose. Shown are representative of three replicates

In CRISPR-based genome editing, it is imperative that the expression of the elements comprising a CRISPR system (i.e., the *cas* gene and small guide RNA) be strictly controlled since high expression levels of Cas proteins are often toxic in a number of bacterial species [28–30]. Additionally, control of gene expression has been proven critical to obtaining the desired phenotype (e.g., high productivity of target metabolite) in metabolic engineering and synthetic biology studies [31, 32]. Therefore, we determined the strength of several promoters in AWRP under a heterotrophic growth condition (Fig. 2): three constitutive promoters P_*fdx*_, P_*pta*_ and P_*trxA*_, which were chosen according to the previous transcriptome study during growth on CO_2_ + H_2_ [19], one putative inducible promoter P_*xyl*_ (upstream of DMR38_09110), and two promoters from *C. acetobutylicum* (P_*thl*_ and P_*ptb*_) [33–35]. Promoters were cloned upstream of *bgaL* from *C. beijerinckii* (Cbei_1236) and a synthetic terminator BBa_B1010 [36, 37]. Upon transformation, the β-galactosidase assay showed that P_*trxA*_ exhibited the highest strength in AWRP among the promoters tested (Fig. 2A). We failed to transform AWRP with the plasmid in which P_*fdx*_ was cloned, indicating that this promoter might not be appropriate for use on multicopy plasmids. Interestingly, P_*thl*_, which had been shown to be strong and approximately four to sixfold higher than P_*ptb*_ in *C. ljungdahlii* [14], was not strong in AWRP: the strength was only 1.5 times higher than that of P_*ptb*_ (Fig. 2A). The P_*xyl*_ promoter showed a strength comparable to P_*trxA*_ upon induction with 10 mM xylose (Fig. 2B). This promoter was found to be slightly leaky under an uninduced condition (Fig. 2A and B), possibly due to the absence of the repressor gene in the plasmid. Although P_*xyl*_ was not stringent, it can be used for a weak promoter for the expression of genetic elements that would be detrimental to growth when expressed under a strong promoter.

### CRISPR/Cas12a-mediated allelic exchange in AWRP

Since episomal plasmids used in clostridia are often unstable without selection pressure, it is desirable to integrate a cassette in strain engineering for industrial purposes. Despite some examples, it has been determined that homologous recombination in clostridial species is difficult with suicide plasmids, as many species do not encode Holliday junction resolvase in their genomes. [38, 39]. Thus, chromosomal manipulation of those bacteria requires tricky methods. For example, in ACE, a promoter-free marker was used for mutant selection, by designing the plasmid so that the marker could be expressed under an active promoter on the chromosome [13, 40]. CRISPR-based genome editing has been widely employed in various clostridial species, including acetogenic strains [41], where the double-strand break (DSB) of the chromosome strongly drives homologous recombination.

As CRISPR/Cas9 has been shown to work effectively in acetogenic strains including *C. ljungdahlii* and *C. autoethanogenum* [14, 15], we cloned *cas9* of *Streptococcus pyogenes* under the control of P_*ptb*_, which was shown to be the weakest among the constitutive promoters tested (see Fig. 2A). However, the resulting plasmid failed to be introduced into AWRP (see the Supporting Information for plasmid construction). This was not due to mutations in the replicon or the *cas9* expression cassette during the cloning process (data not shown). Furthermore, this outcome was quite unexpected in that P_*thl*_ was used for *cas9* expression in *C. ljungdahlii* [14]. This result indicated that the expression level of *cas9* was still too strong in AWRP despite its close relationship to *C. ljungdahlii* and *C. autoethanogenum*.

**Fig. 3:**
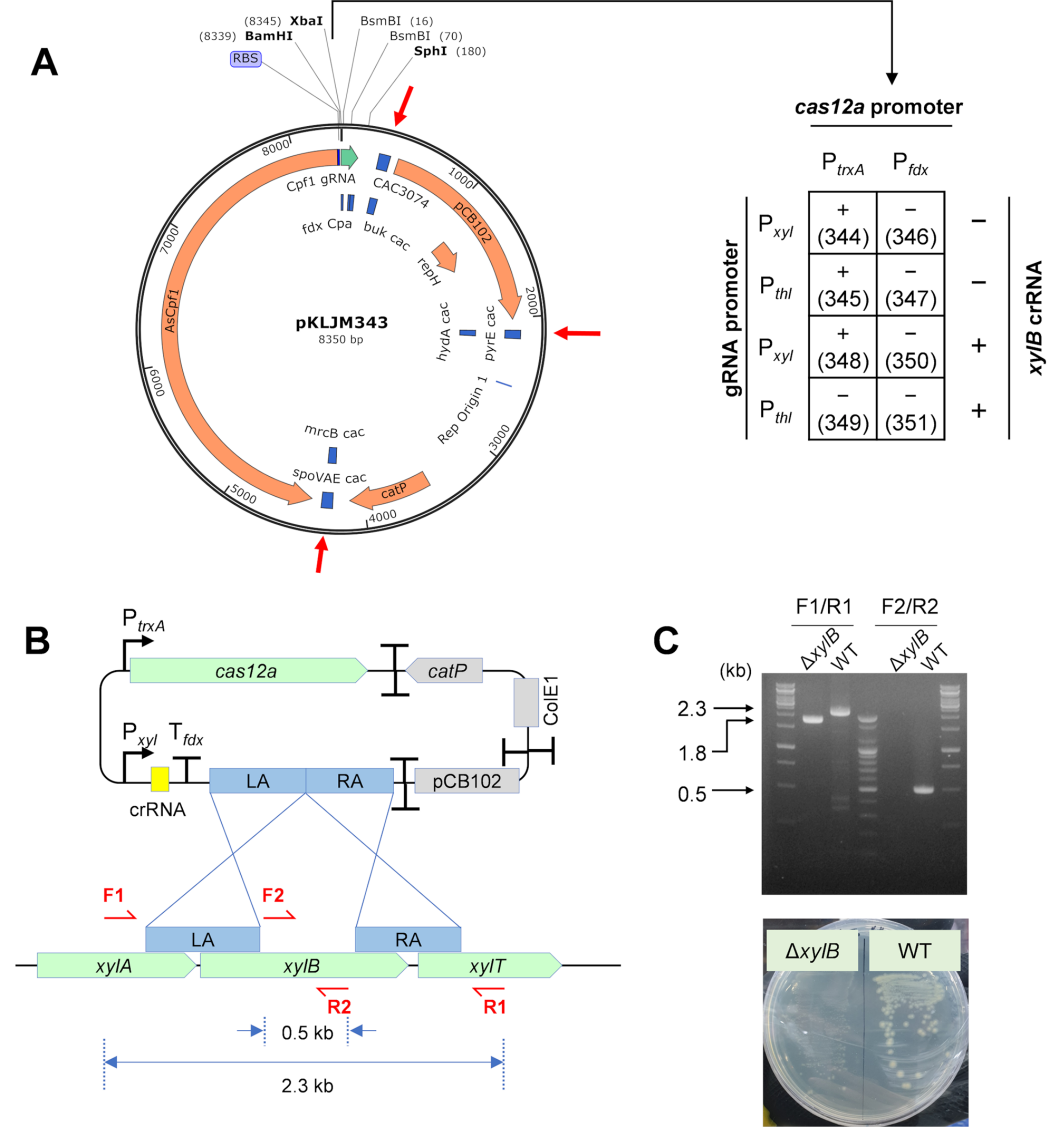
Genome editing with CRISPR/Cas12a in *Clostridium* sp. AWRP. (**A**) Construction of CRISPR/Cas12a genome-editing plasmids that differ in the promoters driving Cas12a and crRNA expression. The combinations of two promoters were assembled in *Bam*HI/*Xba*I-digested pKLJM343. Bidirectional transcriptional terminators (red arrows) of *Clostridium acetobutylicum* were cloned to prevent possible transcriptional interference between the elements on the plasmid. The table on the right shows the results of transformation and genome editing upon the introduction of various plasmid constructs. The plus (+) signs in the table indicate the occurrence of transformed colonies, and the minus signs (–) indicate no transformed colonies were observed. The numbers in brackets indicate the plasmid number (see Table S1). (**B**) Schematic diagram of the *xylB* deletion. Primer binding sites are indicated by red, half arrows. LA and RA indicate two homologous arms for the disruption of *xylB*. Primer sequences are available in Table S2. (**C**) Confirmation of the *xylB* mutant by colony PCR (top) and growth on LBFX medium (bottom). LBFX medium was prepared the same as LBFA medium except that 5 g L^− 1^ fructose was replaced with 5 g L^− 1^ xylose

Instead of searching for a weaker promoter, we decided to employ CRISPR/Cas12a (a.k.a. Cpf1) from *Acidaminococcus* sp. BV3L6 (AsCas12a), which is known to exhibit lower basal toxicity than Cas9 from *S. pyogenes* (SpCas9) [42, 43] (Fig. 3). Furthermore, AsCas12a recognizes the adjacent AT-rich protospacer motif 5′-TTTV-3′ (PAM), which may be more advantageous than Cas9 in the genome editing of AT-rich microbes, including clostridia [44]. Subsequently, we cloned *cas12a* from pDEST-hisMBP-AsCpf1-EC [45]. Furthermore, we constructed a new shuttle plasmid that was partitioned with three bidirectional terminators, to minimize potential transcriptional interference between the elements (Fig. 3A; see also Table S1 for pKLJM210). We observed that the plasmid carrying the AsCas12a expression cassette, but not Cas9, was introduced AWRP under the control of the same P_*ptb*_, indicating that AsCas12a may be more suitable in AWRP (Fig. S1). Although we attempted to edit the AWRP genome after cloning a CRISPR RNA (crRNA) expression cassette under the control of P_*pta*_ into the AsCas12a expression plasmid, we could not observe genome editing from transformed colonies (data not shown). This result indicates that crRNA or AsCas12a expression should be increased.

Next, we tested a couple of combinations of promoters to control the expression of the elements, along with a nontargeting control sequence or *xylB*-targeting crRNA (Fig. 3A). With the control sequence, the attempts to express *cas12a* with the strong P_*fdx*_ promoter resulted in unsuccessful transformations, which again proves that this promoter is not suitable for genetic modifications in AWRP. When P_*trxA*_ was used for *cas12a* expression, transformants were observed regardless of the promoter used for crRNA expression (Fig. 3A). Subsequently, the two plasmids were subjected to genome editing with crRNA targeting *xylB* (DMR38_09115; see Fig. 3B). No transformant was observed when the crRNA was expressed under the control of P_*thl*_ (Fig. 3A). In contrast, a few colonies with slower growth than typical transformants with nontargeting plasmids were observed when P_*xyl*_ was employed for crRNA expression. In the first screening, one of the colonies was found to be a mixture of wild-type and edited cells (i.e., deletion of *xylB*). When the colony was streaked onto the solid media supplemented with 10 mM xylose, we successfully obtained colonies with the edited genome (Fig. 3C). The resulting strain showed a growth defect in xylose when compared to the wild-type AWRP, confirming *xylB* deletion (Fig. 3C). Similarly, we construct a *pyrE* mutant with the AsCas12a plasmid (Fig. S2). In conclusion, this result indicates that strong expression of both crRNA and AsCas12a can cause unsuccessful transformation in AWRP, possibly due to the failure to repair a DSB within the transformation recovery process despite the presence of homologous arms. In *C. ljungdahlii*, a similar result was observed in *pyrE* deletion using Cas12a from *Francisella tularensis*, which was overcome using an optimized transformation protocol [16]. In this study, the use of the inducible *xyl* promoter facilitated the growth of transformants on selective media with an unoptimized transformation protocol, although this might sacrifice editing efficiencies in the first screening stage. However, once transformants were obtained, genome-edited colonies could be easily obtained in the second screening with xylose induction.

### Multiple deletions of gene clusters

**Fig. 4:**
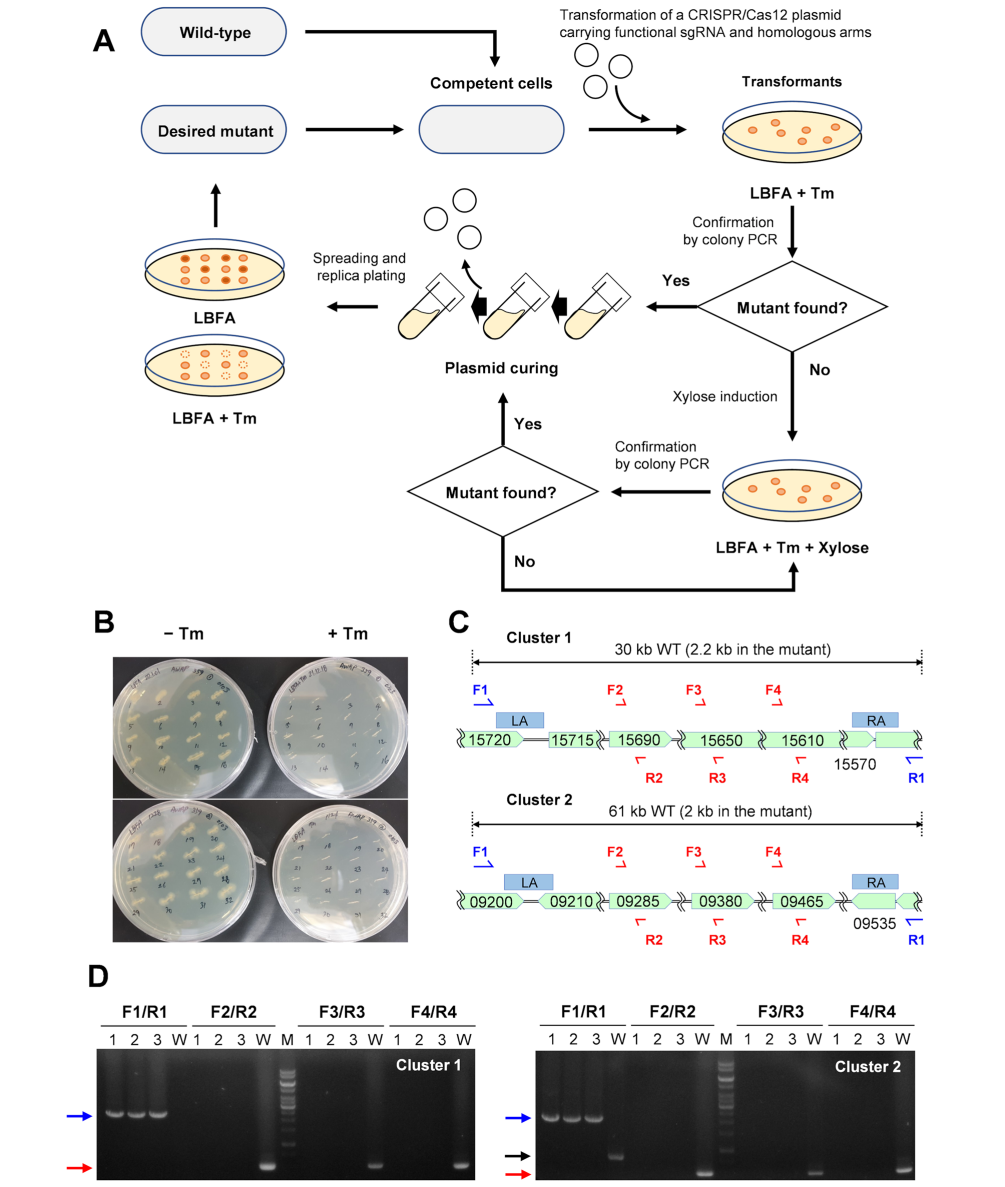
Construction of a double mutant of prophage clusters. (**A**) Schematic diagram of the iterative procedure of gene knockout with the CRISPR/Cas12 system. (**B**) Curing of pKLJM359 after deletion of the first prophage group (DMR38_15715-15570). The replica was done after three serial transfers on LBFA (2% inoculum) in the absence of antibiotic pressure. (**C**) Schematic diagram of two prophage groups. Two homologous arms (LA and RA) are shown in blue boxes, and the binding sites of the verification primers (F1 to F4 and R1 to R4) are indicated in half arrows, respectively. Primer sequences are available in Table S2. (**D**) Verification of genotype of the double deletion mutants. Three colonies were chosen after curing the second target plasmid. The blue arrows indicate the size of the amplified PCR products with F1/R1 (as indicated in **C**). The black arrow indicates a nonspecific PCR product from the wild-type genomic DNA amplified with F1/R1

In recent metabolic engineering and synthetic biology studies, it has been essential to manipulate multiple sites on the chromosome for gene knockout or knock-in, to achieve a desired phenotype. CRISPR/Cas genome editing has been shown to be a powerful tool in model strains *C. ljungdahlii* and *C. autoethanogenum*, for its applications to multiplexed gene knockdown (CRISPRi) [46] and base editing [47], but multiple chromosomal manipulations with a CRISPR system have hardly been demonstrated [48]. Since we observed DSB-induced homologous recombination in AWRP, we examined whether the system can be used for multiple-round genetic manipulations. To this end, we targeted two prophage clusters in the AWRP genome, DMR38_15570-15715 and DMR38_09210–09530 (Fig. 4); transcription of the former had been observed in our previous study [19]. Although the expected phenotypes of elimination are not clear, these clusters deserve to be deleted in terms of strain stability; deletion of the clusters of prophages was shown to result in enhanced stability of butyl ester production in a metabolically engineered *C. saccharoperbutylacetonicum* [49].

First, we constructed a plasmid targeting the former prophage cluster (pKLJM359; Table S1), and a single knockout mutant was successfully isolated after transformation (Table S1). Since the functional replicon is contained in the genome editing plasmid, one prerequisite for the next round of manipulation is that the plasmid can be easily cured after the elimination of the selection pressure so that another targeting plasmid can be introduced to the mutant (Fig. 4A). Interestingly, the CRISPR/Cas12a plasmid was quite unstable and cured cells could be found after three serial transfers in the absence of antibiotic pressure (Fig. 4B). Then, one of the cured mutants was subjected to the transformation with the second targeting plasmid pKLJM361 (Table S1), and a double mutant could be obtained with the same screening procedure. Verification by colony PCR indeed indicated that both prophage clusters were eliminated (Fig. 4C and D). We have also been able to remove the second plasmid of the double mutant through several serial transfers, indicating that this process is virtually iterative.

## Conclusions

In this study, genetic modification procedures were established, which included shuttle plasmid transformation and chromosomal manipulation through CRISPR/Cas12. Differences in DNA methylation and promoter strength from model strains made it difficult to implement the previously developed procedures for model strains. It was found that the elimination of Dam and Dcm methylation was critical to obtaining sufficient transformation efficiencies for AWRP. Upon the establishment of the shuttle plasmid and the transformation protocol, CRISPR/Cas12a genome editing could be successfully employed for AWRP chromosomal manipulation of AWRP. With this tool, we obtained scar- and marker-free mutants, including not only single knockout mutants but also a mutant carrying multiple chromosomal deletions. Along with the promoters characterized, the tools developed in this study now allow scientific and synthetic biological studies to improve the fermentation of syngas, which often require several genetic manipulations. Furthermore, the system can be further modified for advanced CRISPR-based genome editing applications such as CRISPRi and multiplexed genome editing in this nonmodel acetogenic bacterium.

## Methods

### Bacterial strains and reagents for cloning

All strains and plasmids used in this study are listed in Table S1. Primers used in this study were synthesized by Bionics (Seoul, Republic of Korea). *Escherichia coli* DH5α and NEB Turbo were used for routine cloning experiments. Plasmids were prepared from a Luria-Bertani (LB)-grown *E. coli* culture using a Qiaprep Spin Miniprep Kit (QIAGEN Korea Ltd., Seoul, South Korea). Restriction endonucleases were purchased from New England Biolabs (Ipswich, MA, USA) and Enzynomics (Daejeon, Republic of Korea). All PCR experiments for the cloning process were performed using Pfu-Forte polymerase (Enzynomics). Colony PCR was performed using AccuPower PCR premix (Bioneer, Daejeon, Republic of Korea). T4 DNA polymerase (New England Biolabs) was used in one-step sequence- and ligation-independent cloning (SLIC), which was routinely used to assemble one or two DNA insert(s) into a linearized plasmid [50].

### Culture condition and medium composition for *Clostridium* sp. AWRP

*Clostridium* sp. AWRP was routinely propagated using LBFA medium, which contained: 5 g L^− 1^ fructose, 10 g L^− 1^ Bacto™ Tryptone (Difco), 5 g L^− 1^ Bacto™ yeast extract, 0.5 g L^− 1^ NaCl (Duchefa), 5 g L^− 1^ sodium acetate trihydrate (Junsei) and 0.5 g L^− 1^ L-cysteine·HCl (Sigma). The precultures were carried out in an anaerobic glove box (Coy Laboratory Products, MI, USA) by inoculating frozen AWRP stock in a test tube containing 5 mL of LBFA medium; all the precultures were grown at 37℃ without shaking. Cultivations with a larger volume were conducted using 125-mL serum bottles tightly sealed with a butyl rubber stopper. The headspace of the bottles was flushed with 99.99% N_2_ gas before autoclaving. RM medium was used for the growth of AWRP on gaseous substrates [17]. AWRP culture on gaseous substrates was performed according to the previous study [17]. All cultures were conducted at 37°C, 180 RPM.

### Conjugal transfer

Conjugation was performed as previously reported with minor modifications [20, 21]; *E. coli* S17-1 was used as the donor strain. The *E. coli* donor strain harboring a shuttle plasmid was aerobically grown on an antibiotic-supplemented LB medium. The overnight culture was taken into an anaerobic chamber, and donor cells were collected from 1 mL of the culture in a 2-mL microcentrifuge tube, by centrifugation at 6,000 × *g* for 1 min. Donor cells were washed once with fresh LBFA medium to remove residual antibiotics and resuspended with 200 µL of an AWRP culture of OD_600_ ~ 1. The resulting suspension was spotted on fresh nonselective LBFA agar plates. After 3 days of mating at 37℃, cells were collected from the mating plates by resuspending in 2 mL of LBFA medium with a sterile spreader. The mixture was spread onto selective LBFA agar plates which were also supplemented with polymyxin B to counterselect an *E. coli* donor (at the final concentration of 100 IU mL^− 1^). To determine the density of the donor cells, the mixture was serially diluted and placed in selective LB agar plates, which were aerobically incubated at 37°C.

### Electrotransformation

Two serum bottles, each containing 100 mL of LBFA medium, were inoculated with 1 mL of vigorously grown preculture and incubated at 37°C, 180 RPM. When the cell density reached an OD_600_ of 0.3–0.4, the cultures were kept on ice for 30 min. The ice-chilled cultures were taken into the anaerobic chamber and transferred to four 50 mL conical tubes. Cells were collected by centrifugation at 4,000 × *g*, room temperature for 10 min. Cells were washed twice with room temperature electroporation medium (0.27 M sucrose in ultrapure water) and finally resuspended with 1 mL of the electroporation medium. Next, 100 µL of cells were mixed with 10 µL of ice-cold plasmid DNA and transferred to ice-chilled electroporation cuvettes (2-mm electrode gap; Bio-Rad, Hercules, USA). After 5 min of ice incubation, each cuvette was subjected to an exponential electric pulse with the parameters 2.5 kV, 100 Ω, and 25 µF. An ECM 630 electroporation system (Harvard Bioscience, Inc., Holliston, USA) was used to deliver an electric pulse. Upon pulse delivery, the cells were immediately transferred to ice and chilled for 2 min. The cells were mixed with 1 mL of LBFA medium and incubated at 37°C for 4 h. After the recovery step, cells were plated onto selective LBFA agar plates supplemented with 5 µg mL^− 1^ of thiamphenicol.

### β-Galactosidase assay

The wild-type and transformed AWRP strains were grown in a test tube containing 5 mL of the LBFA medium. For each strain, serum bottles containing 100 mL of LBFA were inoculated with 2% inoculum from the overnight culture. The bottles were then incubated at 37°C, 180 RPM until the cell densities reached ca. OD_600_ of 0.5. Cells equivalent to 20 OD_600_ × mL were harvested at 2,500 × *g*, 4°C for 10 min and washed once in the assay buffer (60 mM Na_2_HPO_4_, 40 mM NaH_2_PO_4_, 10 mM KCl, 1 mM MgSO_4_, and 2.7 mL L^− 1^ 2-mercaptoethanol). Finally, the collected cells were resuspended in 1 mL of the buffer. The crude extract was prepared from the resuspension by sonication followed by centrifugation at 12,000 × *g*, 4°C for 10 min. Total protein concentrations were estimated by the Bradford assay using Bio-Rad Protein Assay (Bio-Rad) according to the manufacturer’s procedures. In order to determine β-galactosidase activities, an aliquot of the crude extract (100 µL) was added to a microcentrifuge tube containing 900 µL of assay buffer supplemented with *o*-nitrophenyl-β-D-galactopyranoside (at a final concentration of 1 mg mL^− 1^). The microtubes were kept in a 37℃ water bath for 5 min. Then 500 µL of 1 M Na_2_CO_3_ solution was added to the reaction mixture to stop the reaction. Next, A_420_ values were measured for each tube, which was converted to the amount of *o*-nitrophenol released (with the extinction coefficient of 0.0045 µM^− 1^ cm^− 1^). One unit of activity was defined as the amount of enzyme that catalyzed the formation of 1 nmol of *o*-nitrophenol per min at 37°C.

### Strain construction with CRISPR/Cas12a

For *xylB* deletion, the CRISPR RNA (crRNA) fragment, which was an annealed product of two complementary oligos (see Tables S2 and S3), was ligated into the *Bsm*BI-digested pKLJM344 (see Fig. 3A and Supporting Information), yielding the plasmid pKLJM351. Two arms for homologous recombination were amplified from AWRP genomic DNA with primer pairs xylB-LA-F/R and xylB-RA-F/xylB-RA-R and assembled together into *Sph*I-digested pKLJM351 via one-step SLIC [50]. Once the crRNA and homologous arms were verified, the plasmid was introduced to *E. coli* JM110 for the preparation of methylation-free plasmid DNA. The prepared DNA was used to transform AWRP as described above. the transformants were subjected to colony PCR with the primer pairs to determine the occurrence of homologous recombination (see Figs. 3B and 4C, and Table S2). If no desired genotype was observed, several transformants were streaked onto a selective LBFA medium supplemented with 10 mM xylose. The resulting colonies were subjected to colony PCR for isolation of the mutant. The *pyrE* mutant was constructed through a similar procedure (see Supporting Methods for details).

### Double knockout of prophage clusters

The plasmid pKLJM359, which targets the first prophage cluster (DMR38_15715-15570), was constructed and transformed into AWRP, and mutant colonies were identified by colony PCR with the primer pairs Phage1-cf-F1/R1 to Phage1-cf-F4/R4 (Table S2). One colony was grown in an LBFA medium without antibiotic supplementation. The culture was transferred twice to a fresh medium (2% inoculum) to enrich the cells cured of the plasmid, and then the final culture was streaked onto agar plates without antibiotic pressure. The cured colonies were identified through replica plating onto the thiamphenicol-supplemented LBFA agar plates. One of the cured clones was subjected to the deletion of the second prophage cluster (DMR38_09210-09530) through the similar procedure with the targeting plasmid pKLJM361.

### Electronic supplementary material

Below is the link to the electronic supplementary material.

Additional file 1: Supplementary Methods; **Fig. S1**. Transformation of unmethylated pKLJM082 and pKLJM083; **Fig. S2**. Schematic diagram of *pyrE* deletion in AWRP and confirmation of the genotype and phenotype; **Table S1**. Strains and plasmids used in this study; **Table S2**. Primers used in this study; **Table S3.** Guide RNA sequences for genome editing; Sequence of the synthesized guide RNA cassette for AsCas12a (cloned in pUC57)
