# Supplementary material for: Development of a genetic engineering toolbox for syngas-utilizing acetogen Clostridium sp. AWRP
Source: Microb Cell Fact. 2024 Jan 3;23:6. doi: 10.1186/s12934-023-02272-2 (PMC10763472; doi:10.1186/s12934-023-02272-2)
Supplement: Supplementary file 1 — Additional file 1: Supplementary Methods; Fig. S1. Transformation of unmethylated pKLJM082 and pKLJM083; Fig. S2. Schematic diagram of pyrE deletion in AWRP and confirmation of the genotype and phenotype; Table S1. Strains and plasmids used in this study; Table S2. Primers used in this study; Table S3. Guide RNA sequences for genome editing; Sequence of the synthesized guide RNA cassette for AsCas12a (cloned in pUC57) [file 12934_2023_2272_MOESM1_ESM.docx]

**Supporting Information for**

**Development of a Genetic Engineering Toolbox for Syngas-Utilizing Acetogen *Clostridium* sp. AWRP**

**Supporting Methods**

**Determination of restriction endonuclease activities in *Clostridium* sp. AWRP**

Protoplast extract of *Clostridium* sp. AWRP was prepared according to the previous protocol [1]. The AWRP cultures were grown onto OD_600_ of 0.4-0.6 on 200 mL of LBFA containing 4 g L^-1^ glycine. The protoplasts were harvested by centrifugation at 5,000 × *g*, 4°C, washed twice in 20 mL of CBM (10% sucrose, 25 mM MgCl_2_, and 25 mM CaCl_2_), and lysed in 4 mL TEMK buffer (4 mM Tris∙Cl, pH 8.0, 10 mM EDTA, 6.6 mM 2-mercaptoethanol, and 25 mM KCl). Debris was removed by centrifugation at 16,000 × *g*, 4°C for 30 min, and the supernatant was stored at -80°C before use. Plasmid digestion with protoplast extract was done at 37°C in a 20-µL volume for 2 h, with 1 µg of pKLJM005 and commercial restriction endonuclease buffers (NEBuffer 1.1, 2.1, 3.1, and CutSmart) and qualitatively assessed with agarose gel electrophoresis.

**Construction of shuttle plasmids pKLJM005 to 008**

The first backbone plasmid pKLJM001 was constructed by ligation of three fragments. The ColE1 origin, for replication in *E. coli*, and the chloramphenicol (Cm)/thiamphenicol (Tm) marker were amplified by PCR from pMTL007c-E2 with the primer pairs ColE1-F/R and catP-F/R, respectively (Table S2). *Bsa*I-digested products of these fragments with ligated together with the annealed oligo pair of pKLJM001-MCS-F and -R. The pKLJM003 plasmid was constructed through Gibson assembly of *Eco*RI-digested pKLJM001 and the fragment containing *traJ*-*oriT* from pMTL007c-E2 amplified with the primer pair traJ-oriT-F/R. Gram-positive replicons pCB102, pAMβ1, pIP404, and pIM13 were amplified from pMTL007c-E2, pMTL500E, pJIR750ai, and pIKM1, respectively. The products were assembled with *Hin*dIII-digested pKLJM003 through one-step SLIC, yielding pKLJM005, 006, 007, and 008, respectively.

**Construction of plasmids for β-galactosidase assay**

Plasmid pKLJM036 was constructed through one-step SLIC by the assembly of pKLJM005 backbone (amplified with 005-inv-F/R; except for the *traJ*-*oriT* region) and the PCR product of the synthetic expression cassette from pUC57-76UTRv1 (amplified with EC-005-F/R). To construct pKLJM064, the *bgaL* gene from *C. beijerinckii* (Cbei_1236) was amplified by PCR with bgaL-F/R. *Bam*HI/*Mlu*I-digested PCR product and pKLJM036 were ligated to assemble into pKLJM064. Plasmids pKSJ102 and 104, harboring P*_trxA_* and P*_thl_* in place of P*_ptb_* in pKLJM064, were created by amplification of these promoters from AWRP and *C. acetobutylicum* genomic DNA with PtrxA-F/R and Pthl-F/R, followed by ligation with *Pst*I/*Bam*HI-digested pKLJM064 backbone. Plasmids pKLJM084 (P*_pta_* from AWRP), 085 (P*_xyl_* from AWRP), and 086 (P*_fdx_* from AWRP) were constructed by amplification of the promoters from AWRP genomic DNA with primer pairs 064-Ppta-F/R, 064-Pxyl-F/R, and 064-Pfdx-F/R, followed by assembly with *Pst*I/*Bam*HI-digested pKLJM064 through one-step SLIC, respectively.

**Construction of plasmids for determination of toxicity from Cas9 and Cas12a expression**

Plasmid pKLJM081 was constructed by inverse PCR of pKLJM036 to insert the *lacO* sequence between the MCS and the P*_ptb_* promoter to lessen the possibly toxicity during cloning process in *E. coli*. Plasmids pKLJM082 and 083 were constructed through one-step SLIC with the cas9 CDS from *S. pyogenes* and Cas12a from *Acidaminococcus* sp.; the CDSs were amplified from pUC57-cas9 and pDEST-hisMBP-AsCpf1-EC with Cas9-081-F/R and Cas12a-081-F/R, respectively.

**Construction of plasmids for CRISPR/Cas12a with various promoter combinations**

The shuttle plasmid pKLJM203 was constructed by cloning three bidirectional terminators from *C. acetobutylicum* into the pKLJM001 plasmid: T*_mrcB/spoVAE_* (amplified with TmrcB-F/R), T*_buk/_*_CA_C3074_ (Tbuk-F/R), and T*_hydA/pyrE_* (ThydA-F/R). Plasmid pKLJM210 was constructed by cloning the pCB102 replicon (amplified with pCB102-203-F/R) into *Eco*RI-digested pKLJM203. The *cas12a* gene was amplified from pDEST-hisMBP-AsCpf1-EC with the primer pair Cas12a-210-F/R, which added an RBS and a stop codon. The resulting product was assembled with *Pst*I/*Bam*HI-digested pKLJM210 through one-step SLIC, resulting in plasmid pKLJM342. The synthetic guide RNA cassette was amplified from pUC57-gRNA with gRNA-342-F/R and ligated with *Xba*I/*Sph*I digested pKLJM342, yielding pKLJM343 (see also Fig. 3A). The two promoters to be tested were assembled together with one-step SLIC. For example, to clone P*_trxA_* for *cas12a* and P*_xyl_* for the crRNA cassette (pKLJM344), the primer pair of PtrxA-343-F/R and the pair of Pxyl-trx-343-F/Pxyl-343-R were used for amplification of the promoters, respectively. When P*_fdx_* was combined to P*_xyl_* (pKLJM346), Pxyl-fdx-343-F was used for amplification in place of Pxyl-trx-343-F. Plasmids pKLJM345 (P*trxA*/P*_thl_*) and 347 (P*_fdx_*/P*_thl_*) were constructed through the same procedure. In pKLJM344 to 347, the 23-nt guide RNA (see Table S3) was prepared by annealing two oligonucleotides, which could be fit of the BsmBI-digested plasmids (see the last section in the Supporting Information for the detailed sequence of the crRNA cassette). For example, *pyrE* targeting crRNA was cloned by annealing of pyrE-gRNA-F and -R. The crRNAs targeting prophage clusters were prepared in a similar manner with primer pairs Phage1-gRNA-F/R and Phage2-gRNA-F/R. Homologous arms were amplified with primer pairs (*i.e.*, pyrE-LA-F/R and pyrE-RA-F/R for *pyrE* deletion) and cloned into *Sph*I-digested a crRNA-containing plasmid (*i.e.*, pKLJM356; Table S1) through one-step SLIC.

**Supporting Figures**


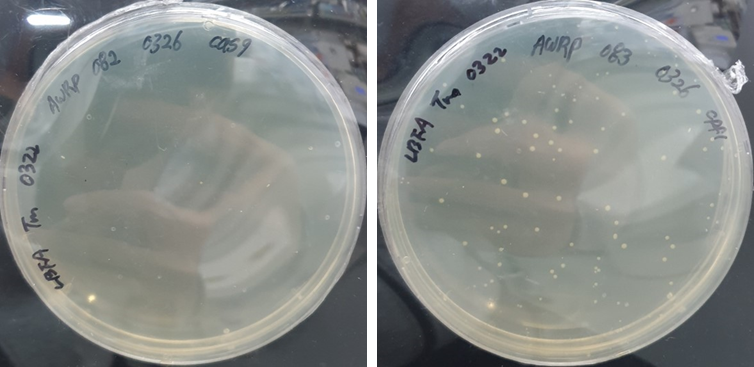


**Fig. S1.** Transformation of unmethylated pKLJM082 (*cas9* under the control of P*_ptb_*; see Table S1) and pKLJM083 (*cas12a* under the control of P*_ptb_*).


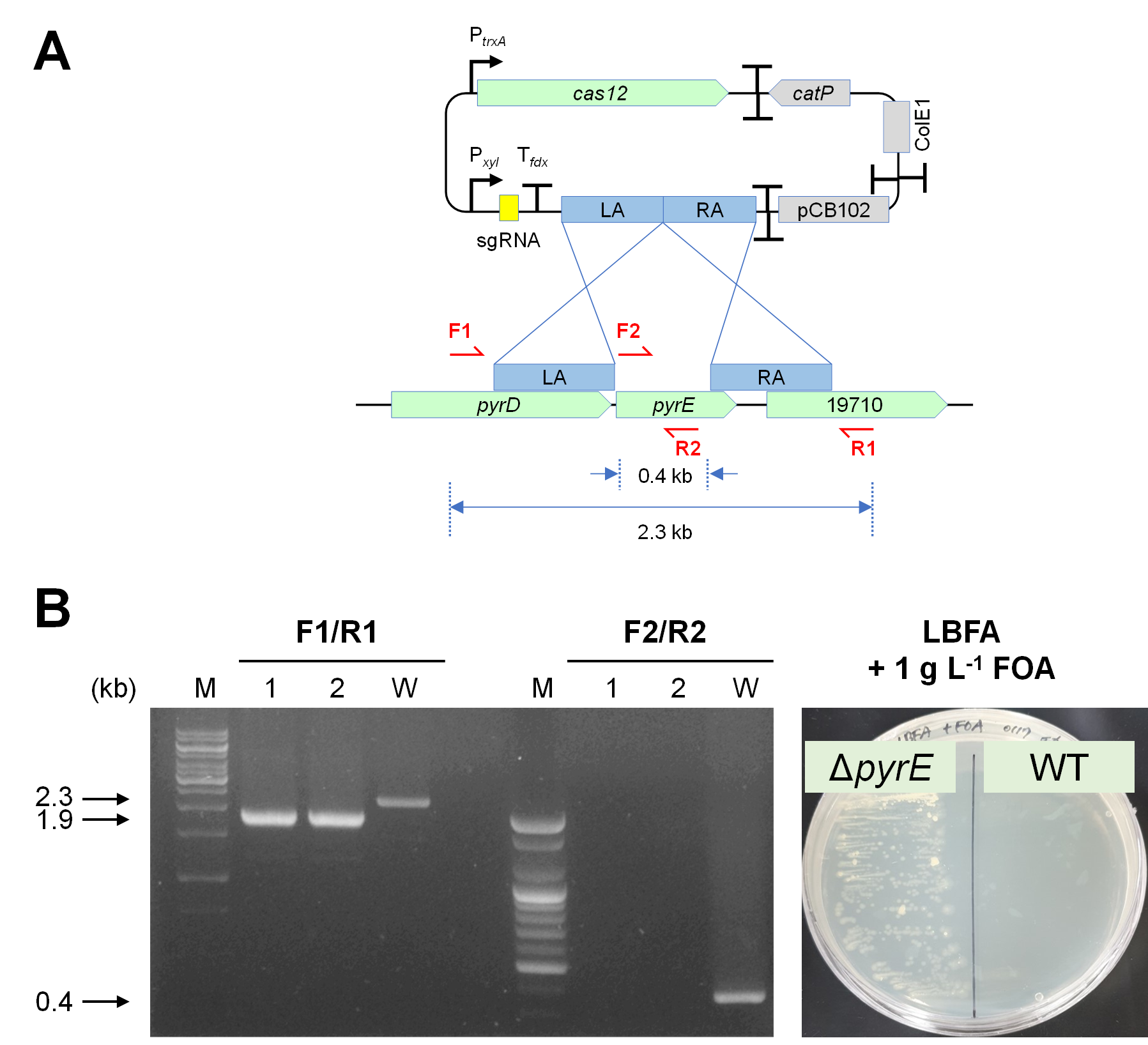


**Fig. S2.** Schematic diagram of *pyrE* deletion in AWRP (A) and confirmation of the genotype and phenotype (B). For phenotypic verification, the wild-type strain and the *pyrE* mutant were grown on LBFA agar supplemented with 1 g L^-1^ fluoroorotic acid (FOA).

**Table S1**. Strains and plasmids used in this study

| Strain/plasmid | Description | Source or reference |
| --- | --- | --- |
| Strain |  |  |
| *Clostridium* sp. AWRP | Wild-type strain | [2] |
| *Clostridium acetobutylicum* ATCC 824 | Wild-type strain; for genomic DNA isolation | KCTC |
| *Clostridium beijerinckii* NCIMB 8052 | Wild-type strain; for genomic DNA isolation | KCTC |
| *E. coli* DH5α | Cloning host; F^–^ *endA1* *glnV*44 *thi*^-1^ *recA1* *relA gyrA*96 *deoR* *nupG* *purB*20 φ80d*lacZ*ΔM15 Δ(*lacZYA*-*argF*)U169, *hsdR*17(r_K_^–^m_K_^+^), λ^–^ | Enzynomics |
| *E. coli* NEB Turbo | F' proA^+^B^+^ lacI^q^ ∆lacZM15 / fhuA2  ∆(lac-proAB)  glnV galK16 galE15  R(zgb-210::Tn10)Tet^S^  endA1 thi-1 ∆(hsdS-mcrB)5 | NEB |
| *E. coli* BL21(DE3) | *E. coli* str. B F^–^ *ompT* *gal* *dcm* *lon* *hsdSB*(r_B_^–^m_B_^–^) λ(DE3 [*lacI* *lacUV5*-*T7p07* *ind1* *sam7* *nin5*]) [*malB*^+^]_K-12_(λ^S^) | NEB |
| *E. coli* JM110 | *rpsL* *thr* *leu* *thi* *lacY* *galK* *galT* *ara* *tonA* *tsx* *dam* *dcm* *glnV44* Δ(*lac*-*proAB*) e14^-^ [F' *traD*36 *proAB*^+^ *lacIq* *lacZ*ΔM15] *hsdR*17(r_K_^-^m_K_^+^) | Agilent |
| *E. coli* S17-1 | Tp^R^ Sm^R^ *recA*, *thi*, *pro*, *hsdR*-M^+^ RP4-2(Km::Tn7 Tc::Mu-1) | Prof. Si Jae Park |
|  |  |  |
| Plasmid |  |  |
| pMTL007c-E2 | Cm/Tm^r^, ColE1 *ori*, pIP404 replicon, ClosTron cassette | Prof. Nigel Minton |
| pMTL500E | Ap^r^, Em^r^, ColE1 *ori*, pAMβ1 replicon | Prof. Si Jae Park |
| pIKM1 | Km^r^, ColE1 *ori*, pIM13 replicon | [3] |
| pJIR750ai | Cm/Tm^r^, pMB1 *ori*, pIP404 replicon, TargeTron cassette | Sigma-Aldrich (Merck) |
| pUC57-cas9 | Ap^r^, a pUC57 derivative containing a synthesized *cas9* gene from *Streptococcus pyogenes* | This study (Bionics, Korea) |
| pUC57-76UTRv1 | Ap^r^, a pUC57 derivative harboring a synthetic expression cassette comprising P_ptb_ from *C. acetobutylicum*, MCS, and the BBa_B1010 terminator [4] | This study (Bionics, Korea) |
| pUC57-gRNA | Ap^r^, a pUC57 derivative containing a synthetic guide RNA (crRNA) cassette for CRISPR/Cas12 from *Acidaminococcus sp.* | This study (Bionics, Korea) |
| pDEST-hisMBP-AsCpf1-EC | Ap^r^, a Cas12 expression cassette with a *E. coli* codon-optimized version of the *cas12* gene from *Acidaminococcus sp.* | [5] |
| pKLJM001 | Cm/Tm^r^, *cat* marker cassette ColE1 *ori* from pMTL007c-E2 | This study |
| pKLJM003 | Cm/Tm^r^, a pKLJM001 derivative with the *oriT*-*traJ* region from pMTL007c-E2 cloned | This study |
| pKLJM005 | Cm/Tm^r^, a pKLJM003 derivative with the pCB102 replicon from pMTL007c-E2 cloned into the *Hin*dIII site | This study |
| pKLJM006 | Cm/Tm^r^, a pKLJM003 derivative with the pAMβ1 replicon cloned from pMTL500E into the *Hin*dIII site | This study |
| pKLJM007 | Cm/Tm^r^, a pKLJM003 derivative with the pIP404 replicon from pJIR750ai cloned through the *Hin*dIII site | This study |
| pKLJM008 | Cm/Tm^r^, a pKLJM003 derivative with the pIM13 replicon from pIKM1 cloned through the *Hin*dIII site | This study |
| pKLJM009 | Ap^r^, a pUC19 derivative harboring the synthetic expression cassette from pUC57-76UTRv1 | This study |
| pKLJM014 | Ap^r^, a pKLJM009 derivative with the *E. coli lacO* sequence inserted between P*_ptb_* and MCS | This study |
| pKLJM036 | Cm/Tm^r^, the synthetic expression cassette from pKLJM009 cloned into pKLJM005; the *oriT-traJ* region excluded | This study |
| pKLJM064 | Cm/Tm^r^, a pKLJM036 derivative with the *bgaL* gene from *C. beijerinckii* cloned downstream of P*_ptb_* | This study |
| pKSJ102 | Cm/Tm^r^, a pKLJM064 derivative with the P*_ptb_* replaced with P*_trxA_* from AWRP | This study |
| pKSJ104 | Cm/Tm^r^, a pKLJM064 derivative with the P*_ptb_* replaced with P*_thl_* from *C. acetobutylicum* | This study |
| pKLJM084 | Cm/Tm^r^, a pKLJM064 derivative with the P*_ptb_* replaced with P*_pta_* from AWRP | This study |
| pKLJM085 | Cm/Tm^r^, a pKLJM064 derivative with the P*_ptb_* replaced with P*_xyl_* from AWRP | This study |
| pKLJM086 | Cm/Tm^r^, a pKLJM064 derivative with the P*_ptb_* replaced with P*_fdx_* from AWRP | This study |
| pKLJM081 | Cm/Tm^r^, a pKLJM036 derivative with the *E. coli* *lacO* sequence cloned downstream of P*_ptb_* | This study |
| pKLJM082 | Cm/Tm^r^, a pKLJM081 derivative with the *S. pyogenes* *cas9* gene cloned | This study |
| pKLJM083 | Cm/Tm^r^, a pKLJM036 derivative with the *Acidaminococcus* sp. *cas12a* gene cloned | This study |
| pKLJM201 | Cm/Tm^r^, a pKLJM001 derivative with the *spoVAE*-*mrcB* bidirectional terminator from *Clostridium acetobutylicum* into the *Hin*dIII site | This study |
| pKLJM202 | Cm/Tm^r^, the *buk*-CA_C3074 bidirectional terminator from *Clostridium acetobutylicum* cloned into *Nco*I/*Eco*RI digested pKLJM201 | This study |
| pKLJM203 | Cm/Tm^r^, the *hydA*-*pyrE* bidirectional terminator from *Clostridium acetobutylicum* cloned into *Eco*RI digested pKLJM202 | This study |
| pKLJM210 | Cm/Tm^r^, a pKLJM203 derivative carrying the pCB102 replicon | This study |
| pKLJM342 | Cm/Tm^r^, the *cas12a* gene cloned into the *Bam*HI/*Pst*I-digested pKLJM210 | This study |
| pKLJM343 | Cm/Tm^r^, the crRNA cassette cloned into the *Xba*I/*Sph*I-digested pKLJM342 | This study |
| pKLJM344 | Cm/Tm^r^, a pKLJM343 derivative, P*_trxA_* for *cas12a* expression, P*_xyl_* for crRNA expression | This study |
| pKLJM345 | Cm/Tm^r^, a pKLJM343 derivative, P*_trxA_* for *cas12a* expression, P*_thl_* for crRNA expression | This study |
| pKLJM346 | Cm/Tm^r^, a pKLJM343 derivative, P*_fdx_* for *cas12a* expression, P*_xyl_* for crRNA expression | This study |
| pKLJM347 | Cm/Tm^r^, a pKLJM343 derivative, P*_trxA_* for *cas12a* expression, P*_thl_* for crRNA expression | This study |
| pKLJM348 | Cm/Tm^r^, a pKLJM344 derivative with *xylB* targeting crRNA | This study |
| pKLJM349 | Cm/Tm^r^, a pKLJM345 derivative with *xylB* targeting crRNA | This study |
| pKLJM350 | Cm/Tm^r^, a pKLJM346 derivative with *xylB* targeting crRNA | This study |
| pKLJM351 | Cm/Tm^r^, a pKLJM347 derivative with *xylB* targeting crRNA | This study |
| pKLJM354 | Cm/Tm^r^, a pKLJM348 derivative with *xylB* homologous arms | This study |
| pKLJM356 | Cm/Tm^r^, a pKLJM344 derivative with *pyrE* targeting crRNA | This study |
| pKLJM357 | Cm/Tm^r^, a pKLJM344 derivative with a crRNA targeting the first prophage in AWRP | This study |
| pKLJM358 | Cm/Tm^r^, a pKLJM356 derivative with *pyrE* homologous arms | This study |
| pKLJM359 | Cm/Tm^r^, a pKLJM357 derivative with homologous arms for deletion of the first prophage in AWRP | This study |
| pKLJM360 | Cm/Tm^r^, a pKLJM344 derivative with a crRNA targeting the second prophage in AWRP | This study |
| pKLJM361 | Cm/Tm^r^, a pKLJM360 derivative with homologous arms for deletion of the second prophage in AWRP | This study |

**Table S2**. Primers used in this study

| Name | Sequence |
| --- | --- |
| ColE1-F | A GGTCTC GAATT CATTAATTGCGTTGCGCT |
| ColE1-R | A GGTCTC A GGCCAGATGGTAAG |
| catP-F | A GGTCTC AGGCC AGTGGGCAAGTTGAAAAATT |
| catP-R | A GGTCTC AAGCT TTAGGGTAACAAAAAACACCG |
| pKLJM001-MCS-F | AGCTTCTGCAGATGGATCCTCTAGACGCGTGCATGCCATGG ACCCGGGCGCCG |
| pKLJM001-MCS-R | AATTCGGCGCCCGGGTCCATGGCATGCACGCGTCTAGAGG ATCCATCTGCAGA |
| traJ-oriT-F | TGGACCCGGGCGCCGAATTC CCTGCTTCGGGGTCAT |
| traJ-oriT-R | TGAGCGCAACGCAATTAATG TCGGTCTTGCCTTGCT |
| pCB102-F | TGTTTTTTGTTACCCTAAAGCTT CTGTCAGACACTTATCACATTAAG |
| pCB102-R | CTAGAGGATCCATCTGCAGA GCCATTATTTTTTTGAACAA |
| pAMb1-F | TGTTTTTTGTTACCCTAAAGCTT CTATTTAATCACTTTGACTAGCAAATAC |
| pAMb1-R | CTAGAGGATCCATCTGCAGA AACTAACTCAACGCTAGTAGTGG |
| pIM13-F | TGTTTTTTGTTACCCTAAAGCTT AATCGCATTTCATAGATTGA |
| pIM13-R | CTAGAGGATCCATCTGCAGA TATGTCTTTTGCGCATTCAC |
| pIP404-F | TGTTTTTTGTTACCCTAAAGCTT GAGTGGGTTACATCGAACTG |
| pIP404-R | CTAGAGGATCCATCTGCAGA CAACGGAAGATGACGCAGA |
| 009-lacO-inv-F | GGATAACAATTT GGATCCTCTAGACGCGTGC |
| 009-lacO-inv-R | GCTCACAATTCC AGTACTTAATTGTACTCATTTATAT |
| 005-inv-F | GTTTTACAAC CATTAATTGC GTTGCGCT |
| 005-inv-R | ATCATGGTCA AGCCATTATT TTTTTGAACAATTG |
| EC-005-F | AATAATGGCT TGACCATGAT TACGCCAAGC |
| EC-005-R | GCAATTAATG GTTGTAAAAC GACGGCCAGT |
| bgaL-F | AAA GGATCC GGTGAAAGTGAGGTAAGAGT |
| bgaL-R | AAA ACGCGT CTCTAGTTCCTATATGTGCGT |
| 064-Ppta-F | GCCAAGCTTGCATGCC TAAAAATAAGATAGCATACTGATTG |
| 064-Ppta-R | CCTCACTTTCACCGGATC TTTAACACAAAATTACACACACT |
| PtrxA-F | AAA CTGCAG TAAAGATAGAGATAAGTCACAAGC |
| PtrxA-R | AAA GGATCC CATAGGGGTATTATTTTTAATTATATT |
| Pthl-F | AAA CTGCAG GAATGAAGTTTCTTATGCACA |
| Pthl-R | AAA GGATCC GTTGT TTAATT ACAACTTAATTATACCCACTATTATTA |
| 064-Pxyl-F | GCCAAGCTTGCATGCC TAGTTATGGTTTTGGGGTATT |
| 064-Pxyl-R | CCTCACTTTCACCGGATC TTGTTAAATTTAGTATAACATCCTATTAA |
| 064-Pfdx-F | GCCAAGCTTGCATGCC TGCAGTATTATAAGGTATTTTCAA |
| 064-Pfdx-R | CCTCACTTTCACCGGATC TAACACCTCCTTAATTTTTAGATAA |
| Cas9-081-F | AGCGGATAACAATTTGGATC GTGGATAAGAAATACTCAATAGG |
| Cas9-081-R | CGCCCGGGTCCATG TTAGTCACCTCCTAGCTGAC |
| Cas12a-081-F | AGCGGATAACAATTTGGATC ATGACACAGTTTGAAGGCTT |
| Cas12a-081-R | CGCCCGGGTCCATG GTTTCTCAGTTCTTGAATGTAG |
| TmrcB-F | GGTGTTTTTTGTTACCCTAAAGCT ACTCAAGTTCTGGAAATAACAATC |
| TmrcB-R | AGGATCCATCTGCAGAAGCTT GTGATGATTTTAATAAAGGGTGTCC |
| Tbuk-F | AGACGCGTGCATGCCATGG AGATATGGTGGAGAAGATGAACT |
| Tbuk-R | CGCAACGCAATTAATGAATTC GCAAATATTGGCATGTGGATT |
| ThydA-F | CATGCCAATATTTGCGAATTC TGGAAAACCAGGTGAAGGAC |
| ThydA-R | CGCAACGCAATTAATGAATT TCAATGGCAAAGTAATCATAGATG |
| pCB102-203-F | CATGCCAATATTTGCGAATT GCCATTATTTTTTTGAACAA |
| pCB102-203-R | TCACCTGGTTTTCCAGAATT CTGTCAGACACTTATCACATTAAG |
| Cas12a-210-F | TGCACGCGTCTAGA GGATCC AGGAGGTATATTAAAC ATGACACAGTTTGAAGGCTT |
| Cas12a-210-R | CTTTATTAAAATCATCACAAGCTTC TTA GTTTCTCAGTTCTTGAATGTAG |
| gRNA-342-F | AAAT TCTAGA TAATTTCTACTCTTGTAGATAGAGAC |
| gRNA-342-R | AAAT GCATGC CACACAGGAAACAGCTAT |
| Pfdx-343-F | GGGATTATGAAGAAACTGTACT |
| Pfdx-343-R | CATGTTTAATATACCTCCTGGATC TAATTTTTAGATAATGTTTTCATCC |
| PtrxA-343-F | GGGATTATGAAGAAACTGTACT |
| PtrxA-343-R | CATGTTTAATATACCTCCTGGATC CCATAGGGGTATTATTTTTAATTAT |
| Pxyl-fdx-343-F | TTGAAAATACCTTATAATACTGCATA TAGTTATGGTTTTGGGGTATTT |
| Pxyl-trx-343-F | ACAGTACAGTTTCTTCATAATCCC TAGTTATGGTTTTGGGGTATTT |
| Pxyl-343-R | GTCTCTATCTACAAGAGTAGAAATTATCTAG TGTTGTTAAATTTAGTATAACATCCT |
| Pthl-fdx-343-F | TTGAAAATACCTTATAATACTGCATA GATGAATTTTCACCTAAAAAGTA |
| Pthl-trx-343-F | ACAGTACAGTTTCTTCATAATCCC GATGAATTTTCACCTAAAAAGTA |
| Pthl-343-R | GTCTCTATCTACAAGAGTAGAAATTATCTAG GTTGT TTAATT ACAACTTAATTATACCCACTATTATTA |
| xylB-gRNA-F | AGAT ACTTGATGCAAATACGACACCAG |
| xylB-gRNA-R | ATTA CTGGTGTCGTATTTGCATCAAGT |
| pyrE-gRNA-F | AGAT GTTGTAAATACATTGAAAGAAGT |
| pyrE-gRNA-R | ATTA ACTTCTTTCAATGTATTTACAAC |
| Phage1-gRNA-F | AGAT CTATTACACCTACCCATAATTTA |
| Phage1-gRNA-R | ATTA TAAATTATGGGTAGGTGTAATAG |
| Phage2-gRNA-F | AGAT GTATAGAGTCATCATATAAATAT |
| Phage2-gRNA-R | ATTA ATATTTATATGATGACTCTATAC |
| xylB-LA-F | CATAGCTGTTTCCTGTGTGG GGTAAGAAGAGCATCTTTTG |
| xylB-LA-R | ATAAGGTGTTCT GACACATCAGATGCAAATTT |
| xylB-RA-F | ATCTGATGTGTC AGAACACCTTATAACGATCC |
| xylB-RA-R | CTCCACCATATCTCCATGG GAATTAATGCTGATGCAGAG |
| pyrE-LA-F | CATAGCTGTTTCCTGTGTGG CCAATTGATAGATATATGCG |
| pyrE-LA-R | TTCTGGATTAC CCTCCTAAATTATTCCTCTG |
| pyrE-RA-F | AATTTAGGAGG GTAATCCAGAAGTTAGGTGG |
| pyrE-RA-R | CTCCACCATATCTCCATGG TATCCGCATTATTCTTTGTC |
| Phage1-LA-F | CATAGCTGTTTCCTGTGTGG ACAAGGTTAGAAATGGGCAGC |
| Phage1-LA-R | TGCTGACTTTGT TTGTAGCTCCTCCCGAAGAC |
| Phage1-RA-F | GAGGAGCTACAA ACAAAGTCAGCACATCACAGA |
| Phage1-RA-R | CTCCACCATATCTCCATGG GCATATTGGTCACAGCCTGG |
| Phage2-LA-F | CATAGCTGTTTCCTGTGTGG TCGGCATCTACTATACATGA |
| Phage2-LA-R | GCATAGATAATTT CTTTTCTAAAGATGTATGACCAC |
| Phage2-RA-F | ATCTTTAGAAAAG AAATTATCTATGCCATTTACTTTT |
| Phage2-RA-R | CTCCACCATATCTCCATGG ACTTAGAAGATTTTAATTCATACTTTT |
| xylB-cf-F1 | AGCACCGGGAGGATTGAACT |
| xylB-cf-R1 | ATCCACGTTTTTCAGCAGGT |
| xylB-cf-F2 | TGATGCTTCTGGAACAGTAA |
| xylB-cf-R2 | CTCTCCTATTAAATAAGGTGAAA |
| pyrE-cf-F1 | GGACTATTTTCTTTCAAGTGC |
| pyrE-cf-R1 | GCTAAATCCTTAGACTTAGTTTG |
| pyrE-cf-F2 | GCTATGGATAATTTAGTTGTAAATAC |
| pyrE-cf-R2 | GCAACCTCTACAGAGGATTT |
| Phage1-cf-F1 | TATTTGGACAGTCTGGTGCT |
| Phage1-cf-R1 | CAAGGGTTCTACTGCAGTAA |
| Phage1-cf-F2 | GCAAGCTGTTAACCAGATTT |
| Phage1-cf-R2 | CCATAAACAGTATCTCCCAA |
| Phage1-cf-F3 | CACATCCAAAATTATTTGCA |
| Phage1-cf-R3 | TGTTTCTCTGACACTTTCAAT |
| Phage1-cf-F4 | ATGCAGATGGAAAGTTAAGG |
| Phage1-cf-R4 | CCCGTTCCAACTATCTTATT |

**Table S3**. Guide RNA sequences for genome editing

| Target | **(PAM)** and gRNA sequence |
| --- | --- |
| *xylB* | **(TTTC)** ACTTGATGCAAATACGACACCAG |
| *pyrE* | **(TTTA)** GTTGTAAATACATTGAAAGAAGT |
| Prophage 1 (DMR38_15570-15715) | **(TTTC)** CTATTACACCTACCCATAATTTA |
| Prophage 2 (DMR38_09210-09530) | **(TTTA)** GTATAGAGTCATCATATAAATAT |

**Sequence of the synthetic guide RNA cassette for AsCas12a (cloned in pUC57)**

| 1  51  101  151  201  251  301 | CAATAAAATA AGTATTAGTG TAGGATTTTT AAATAGAGTA TCTATTTTCA  GATTAAATTT TTAATTATTT GATTTAAATT GTATAATATT TAGTAAAGTA  TTGACTAGTA AAATTTTGTG ACACTTTAAT TTGTGAAATT TCTTAGCTAA  AGTTATATTT TTGAATAATT TTTATTGAAA AATACAACTA AAAAGGATTA  TAGTATAAGT GTGTGTAATT AATTTCTACT CTTGT***AGAT***A GAGACGACCA  TACTATACAC TACATTAAAT TATGTTTCTT GCCGTCTCT***T AAT***TTCTACT  CTTGTAGATA AAAATAAGAA GCCTGCAAAT GCAGGCTTCT TATTTTTA |
| --- | --- |

: putative -35 and -10 sites of the AWRP P*_pta_*

: direct repeats of crRNA for AsCas12a

: *Bsm*BI recognition sequence (cleavage site sequences are italicized)

: ferredoxin terminator from *Clostridium pasteurianum* [6]

The underlined sequence indicates the actual region used in the promoter replacement experiment for crRNA and Cas12a (see Fig. 3A)

**References**

1. Mermelstein LD, Welker NE, Bennett GN, Papoutsakis ET. Expression of cloned homologous fermentative genes in *Clostridium acetobutylicum* ATCC 824. Nat Biotechnol. 1992;10:190-5.

2. Lee J, Lee JW, Chae CG, Kwon SJ, Kim YJ, Lee J-H, et al. Domestication of the novel alcohologenic acetogen *Clostridium* sp. AWRP: From isolation to characterization for syngas fermentation. Biotechnol Biofuels Bioprod. 2019;12:228.

3. Mai V, Lorenz WW, Wiegel J. Transformation of *Thermoanaerobacterium* sp. strain JW/SL-YS485 with plasmid pIKM1 conferring kanamycin resistance. FEMS Microbiol Lett. 1997;148:163-7.

4. Lee J, Jang Y-S, Papoutsakis ET, Lee SY. Stable and enhanced gene expression in *Clostridium acetobutylicum* using synthetic untranslated regions with a stem-loop. J Biotechnol. 2016;230:40-3.

5. Hur JK, Kim K, Been KW, Baek G, Ye S, Hur JW, et al. Targeted mutagenesis in mice by electroporation of Cpf1 ribonucleoproteins. Nat Biotechnol. 2016;34:807-8.

6. Heap JT, Pennington OJ, Cartman ST, Minton NP. A modular system for *Clostridium* shuttle plasmids. J Microbiol Methods. 2009;78:79-85.
